# Supplementary material for: Age-associated differences in mucosal and systemic host responses to SARS-CoV-2 infection
Source: Nat Commun. 2025 Mar 10;16:2383. doi: 10.1038/s41467-025-57655-3 (PMC11894178; doi:10.1038/s41467-025-57655-3)
Supplement: Supplementary file 1 — Supplementary Information [file 41467_2025_57655_MOESM1_ESM.pdf]

SUPPLEMENTARY INFORMATION

Age-associated differences in mucosal and systemic host responses to SARS-CoV-2 infection

Authors: Jillian H. Hurst[1,2,†], Aditya A. Mohan[3,†], Trisha Dalapati[4], Ian A. George[5], Jhoanna N. Aquino[1], Debra J. Lugo[1], Trevor S. Pfeiffer[1], Javier Rodriguez[6], Alexandre T. Rotta[7], Nicholas A. Turner[8], Thomas W. Burke[8,9], Micah T. McClain[8,9,10], Ricardo Henao[11,12], C. Todd DeMarco[13], Raul Louzao[13], Thomas N. Denny[13], Kyle M. Walsh[2,14], Zhaohui Xu[15], Asuncion Mejias[15], Octavio Ramilo[15], Christopher W. Woods[8,9,10,13], Matthew S. Kelly[1*]

Affiliations:

[1]Department of Pediatrics, Division of Infectious Diseases, Duke University School of Medicine; Durham, NC, USA
[2]Children's Health and Discovery Institute, Department of Pediatrics, Duke University School of Medicine; Durham, NC, USA
[3]Department of Biomedical Engineering, Duke University School of Medicine; Durham, NC, USA
[4]Department of Molecular Genetics and Microbiology, Duke University School of Medicine; Durham, NC, USA
[5]Duke University School of Medicine; Durham, NC, USA
[6]Children's Clinical Research Unit, Department of Pediatrics, Duke University School of Medicine; Durham, NC, USA
[7]Department of Pediatrics, Division of Pediatric Critical Care Medicine, Duke University School of Medicine; Durham, NC, USA
[8]Department of Medicine, Division of Infectious Diseases, Duke University School of Medicine; Durham, NC, USA
[9]Center for Infectious Disease Diagnostics and Innovation, Duke University School of Medicine; Durham, NC, USA
[10]Durham Veterans Affairs Medical Center; Durham, NC, USA
[11]Department of Biostatistics and Informatics, Duke University; Durham, NC, USA
[12]Duke Clinical Research Institute, Duke University School of Medicine; Durham, NC, USA
[13]Duke Human Vaccine Institute, Duke University School of Medicine; Durham, NC, USA
[14]Department of Neurosurgery, Duke University School of Medicine; Durham, NC, USA
[15]Department of Infectious Diseases, St. Jude Children's Research Hospital; Memphis, TN, USA
[†]These authors contributed equally to this work.

Supplementary Figures

Fig. S1. Differential expression of individual genes among SARS-CoV-2-uninfected pediatric subjects by age group
Fig. S2. Imputed sample immune cell proportions in children, adolescents, and adults with SARS-CoV-2 infection and uninfected controls
Fig. S3. Differential expression of individual genes in the upper respiratory tract associated with SARS-CoV-2 infection among children and adolescents
Fig. S4. Differential expression of individual genes in peripheral blood associated with SARS-CoV-2 infection among children, adolescents, and adults

Supplementary Tables

Supplementary Table 1. Characteristics of the study population by SARS-CoV-2 infection status
Supplementary Table 2. SARS-CoV-2 lineages identified by genomic sequencing of nasopharyngeal samples (n=28)

Fig. S1. Differential expression of individual genes among SARS-CoV-2-uninfected pediatric subjects by age group. Volcano plots are shown comparing the transcriptional profiles of upper respiratory (**a.**) and peripheral blood (**b.**) samples from SARS-CoV-2-uninfected young children (0-5 years), school-age children (6-13 years), adolescents (14-20 years), and adults (≥21 years, peripheral blood only). For each comparison, the age group listed first represents the group of interest while the age group listed second is the reference group. Differentially expressed genes are colored red (upregulated in the age group of interest) or blue (downregulated in the age group of interest). When applicable, the 10 most differentially upregulated and downregulated genes based on log2-fold change are labeled. All analyses were adjusted for sex, sequencing batch, and imputed sample immune cell proportions (peripheral blood samples only). Source data are provided as a Source Data file.

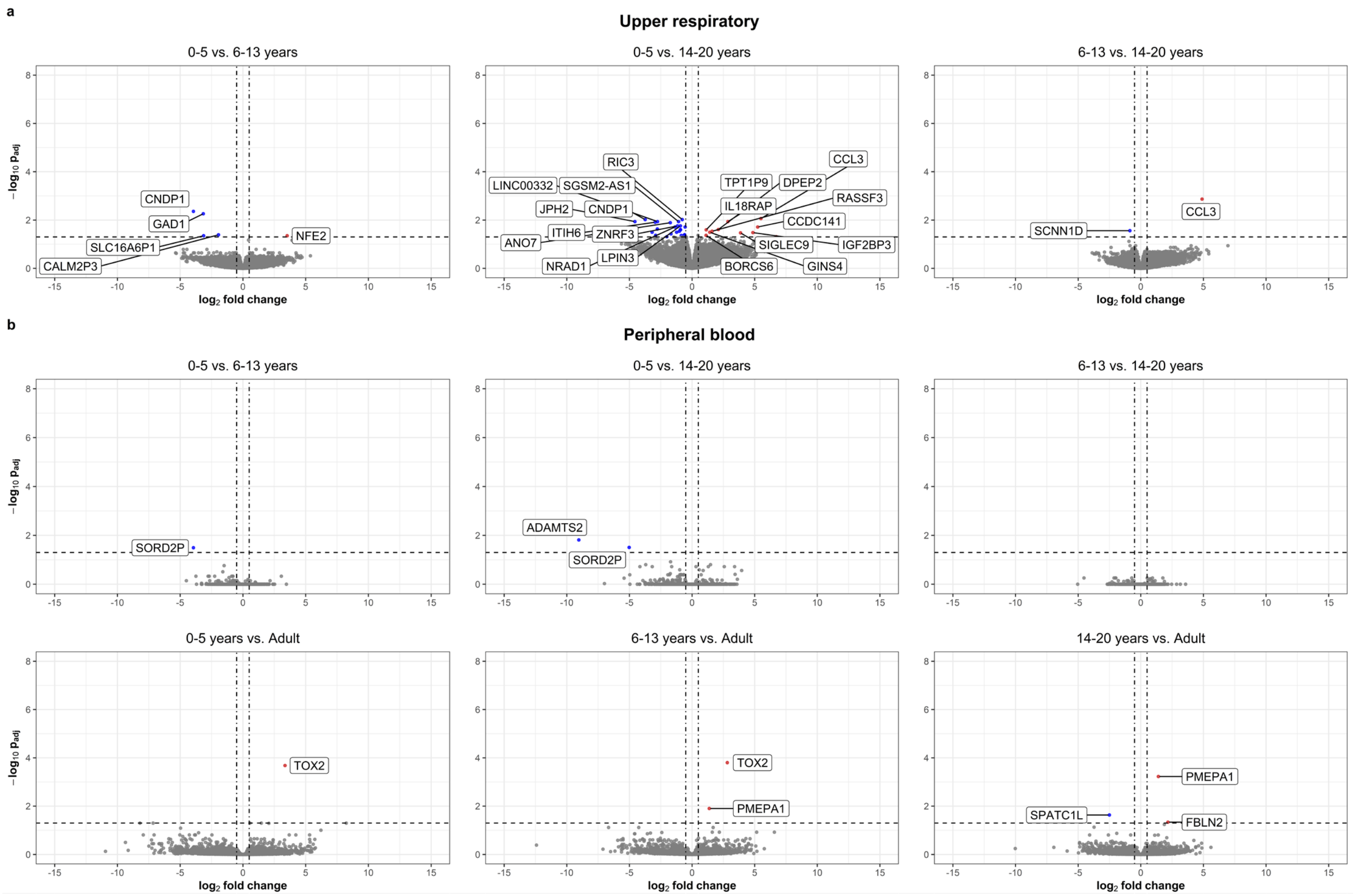

Fig. S2. Imputed sample immune cell proportions in children, adolescents, and adults with SARS-CoV-2 infection and uninfected controls. Bulk RNA sequencing was used to compare the transcriptional profiles of SARS-CoV-2-infected and uninfected young children (0-5 years), school-age children (6-13 years), adolescents (14-20 years) and adults (≥21 years, peripheral blood only). Box and whisker plots depict proportions of immune cell populations imputed using CIBERSORT for upper respiratory (**a.**) and peripheral blood (**b.**) samples. Note that the proportions of cell types predicted by CIBERSORT do not reflect their absolute proportions within a given sample type. Lines splitting the boxes correspond to median values while box edges represent the 25th and 75th percentiles with outliers shown as single points. Data for SARS-CoV-2-infected and uninfected subjects are shown in blue and red, respectively. Proportions of immune cell populations were compared by SARS-CoV-2 status using beta regression, with all analyses adjusted for age (modeled as a continuous variable) and corrected for multiple comparisons (*, $p_{adj}<0.05$; **, $p_{adj}<0.01$; ***, $p_{adj}<0.001$; ****, $p_{adj}<0.0001$). Only immune cell populations identified in at least 25% of samples are shown. Source data are provided as a Source Data file.

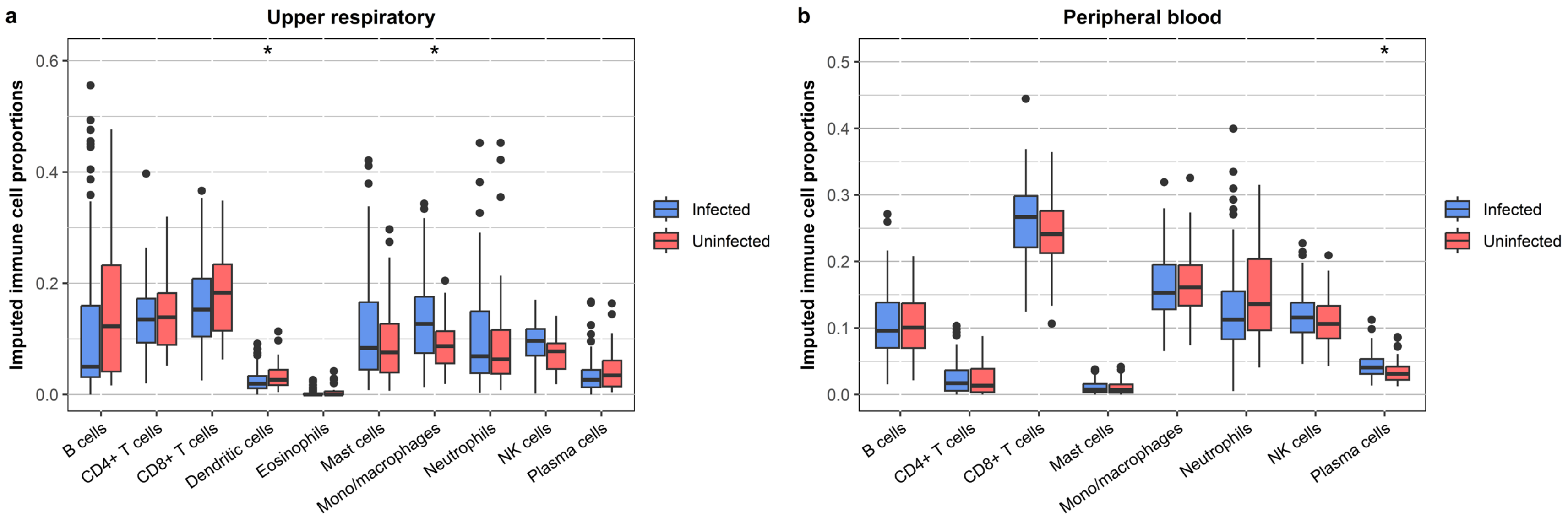

Fig. S3. Differential expression of individual genes in the upper respiratory tract associated with SARS-CoV-2 infection among children and adolescents. Bulk RNA sequencing was used to compare the upper respiratory transcriptional profiles of young children (0-5 years), school-age children (6-13 years), and adolescents (14-20 years) by SARS-CoV-2 infection status. Volcano plots are shown depicting differential expression of genes among SARS-CoV-2-infected subjects relative to uninfected subjects by age group ($p_{adj}<0.05$). When applicable, the 10 most differentially upregulated and downregulated genes based on log2-fold change are labeled. All analyses were adjusted for sex and sequencing batch. Source data are provided as a Source Data file.

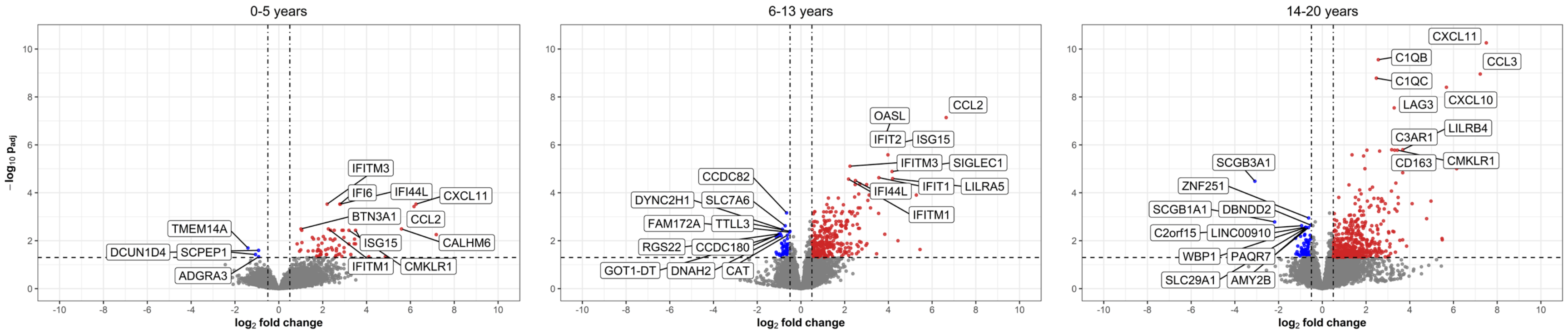

Fig. S4. Differential expression of individual genes in peripheral blood associated with SARS-CoV-2 infection among children, adolescents, and adults. Bulk RNA sequencing was used to compare the peripheral blood transcriptional profiles of young children (0-5 years), school-age children (6-13 years), adolescents (14-20 years), and adults (≥21 years) by SARS-CoV-2 infection status. Volcano plots are shown depicting differential expression of genes among SARS-CoV-2-infected subjects relative to uninfected subjects in the same age group ($p_{adj}<0.05$). The 10 most differentially upregulated and downregulated genes based on log2-fold change are labeled. All analyses were adjusted for sex, sequencing batch, and imputed sample immune cell proportions. Source data are provided as a Source Data file.

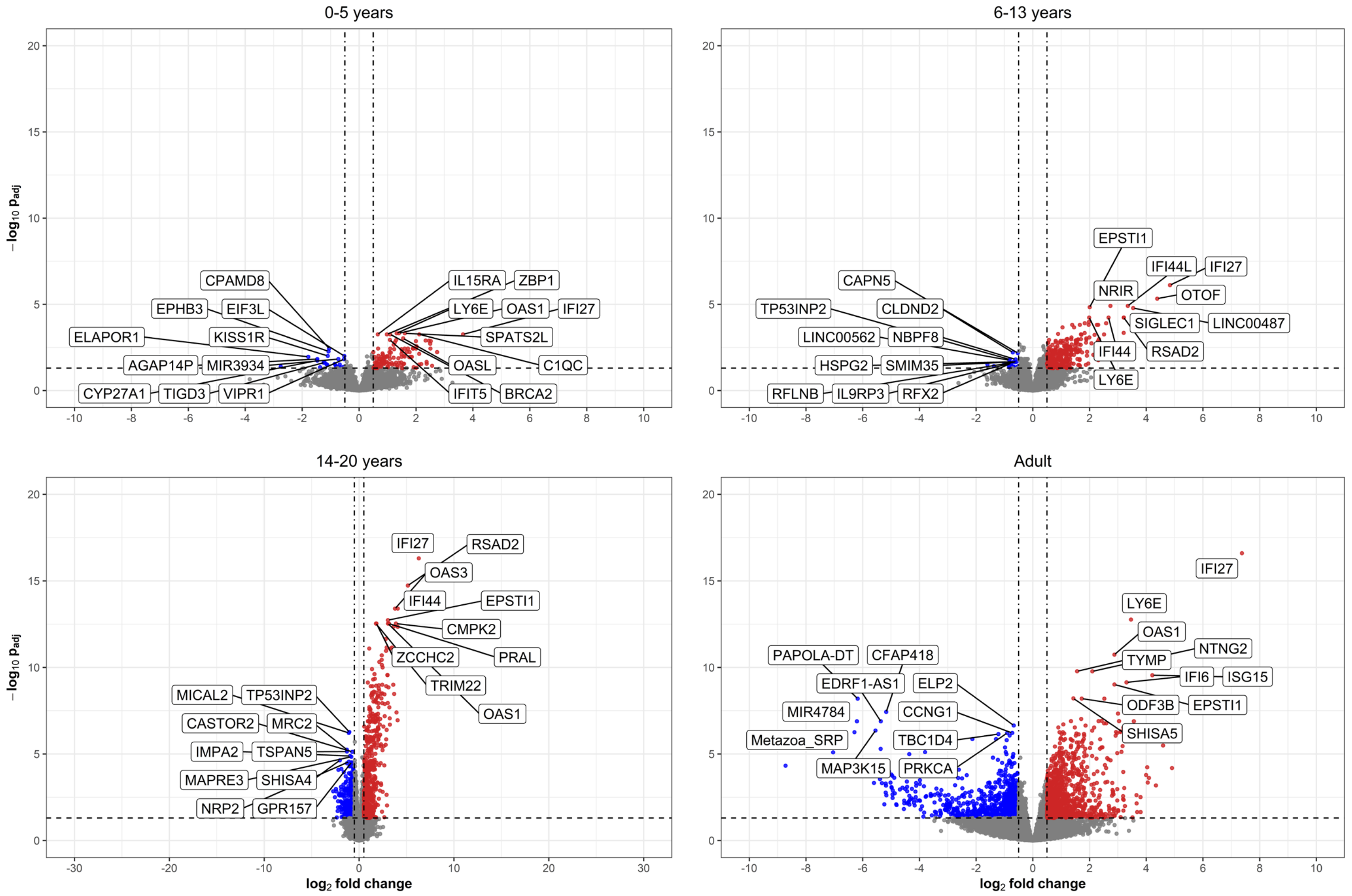

Supplementary Table 1. Characteristics of the study population by SARS-CoV-2 infection status

Characteristics		SARS-CoV-2-infected (n=137)	Healthy (n=65)	*p*
**Young children (0-5 years)**		**n=29**	**n=10**	
	Median [IQR] age, years	2.8 [1.4, 4.8]	3.7 [2.5, 4.8]	0.55
	Female sex	20 (69%)	5 (50%)	0.45
	Obesity†	4 (22%)	3 (38%)	0.64
	Other comorbidities	1 (3%)	2 (20%)	0.16
**School-age children (6-13 years)**		**n=43**	**n=22**	
	Median [IQR] age, years	10.5 [8.5, 12.4]	9.5 [8.0, 11.2]	0.26
	Female sex	20 (47%)	12 (54%)	0.61
	Obesity	14 (33%)	4 (18%)	0.26
	Other comorbidities	7 (16%)	7 (32%)	0.20
**Adolescents (14-20 years)**		**n=39**	**n=17**	
	Median [IQR] age, years	17.3 [15.7, 18.6]	16.5 [14.9, 17.9]	0.33
	Female sex	20 (51%)	10 (59%)	0.77
	Obesity	13 (33%)	5 (29%)	>0.99
	Other comorbidities	7 (18%)	3 (18%)	>0.99
**Adults (≥21 years)**		**n=26**	**n=16**	
	Median [IQR] age, years	46.0 [35.0, 53.2]	47.6 [44.4, 54.0]	0.25
	Female sex	13 (50%)	8 (50%)	>0.99
	Obesity†	5 (24%)	1 (11%)	0.64
	Other comorbidities	14 (64%)	3 (19%)	0.009

†Children <2 years of age were excluded from these analyses; obesity data were missing from 11 adults

Supplementary Table 2. SARS-CoV-2 lineages identified by genomic sequencing of nasopharyngeal samples (n=28)

Viral lineage	Number of genomes
B.1	14
B.1.1	3
B.1.1.231	2
B.1.1.304	1
B.1.1.110.3	1
B.1.2	2
B.1.289	1
B.1.328	1
B.1.332	2
B.1.498	1
