# Supplementary material for: Age-associated differences in mucosal and systemic host responses to SARS-CoV-2 infection
Source: Nat Commun. 2025 Mar 10;16:2383. doi: 10.1038/s41467-025-57655-3 (PMC11894178; doi:10.1038/s41467-025-57655-3)
Supplement: Supplementary file 2 — Description of Additional Supplementary Files [file 41467_2025_57655_MOESM2_ESM.pdf]

Description of Additional Supplementary Files

Supplementary Data 1. Output from fast gene set enrichment analysis of immune module expression in upper respiratory tract samples from healthy subjects.

Supplementary Data 2. Output from fast gene set enrichment analysis of immune module expression in peripheral blood samples from healthy subjects.

Supplementary Data 3. Output from fast gene set enrichment analysis of immune modules in upper respiratory and peripheral blood samples by SARS-CoV-2 infection status.

Supplementary Data 4. Output from fast gene set enrichment analysis of immune modules in upper respiratory samples by SARS-CoV-2 infection status within specific age groups.

Supplementary Data 5. Output from fast gene set enrichment analysis of immune modules in peripheral blood samples by SARS-CoV-2 infection status within specific age groups.

Supplementary Data 6. Output from age-adjusted gene set enrichment analysis of immune modules in upper respiratory samples from SARS-CoV-2-infected participants by symptom presence.

Supplementary Data 7. Output from age-adjusted gene set enrichment analysis of immune modules in peripheral blood samples from SARS-CoV-2-infected participants by symptom presence.

Supplementary Data 8. Correlations between expression of immune modules in paired upper respiratory and peripheral blood samples from the same individual within pediatric age groups.

Supplementary Data 9. Gene modules used for gene set enrichment analysis were based on modules defined by the NanoString nCounter® Host Response Panel. A full list of the genes included in each module is provided.
