# Supplementary material for: Age-associated differences in mucosal and systemic host responses to SARS-CoV-2 infection
Source: Nat Commun. 2025 Mar 10;16:2383. doi: 10.1038/s41467-025-57655-3 (PMC11894178; doi:10.1038/s41467-025-57655-3)
Supplement: Supplementary file 12 — Reporting Summary [file 41467_2025_57655_MOESM12_ESM.pdf]

nature portfolio

Corresponding author(s): Matthew S. Kelly, MD, MPH

Last updated by author(s): Feb 17, 2025

Reporting Summary

Nature Portfolio wishes to improve the reproducibility of the work that we publish. This form provides structure for consistency and transparency in reporting. For further information on Nature Portfolio policies, see our Editorial Policies and the Editorial Policy Checklist.

Statistics

For all statistical analyses, confirm that the following items are present in the figure legend, table legend, main text, or Methods section.

n/a	Confirmed	
☐	☒	The exact sample size (*n*) for each experimental group/condition, given as a discrete number and unit of measurement
☐	☒	A statement on whether measurements were taken from distinct samples or whether the same sample was measured repeatedly
☐	☒	The statistical test(s) used AND whether they are one- or two-sided<br>*Only common tests should be described solely by name; describe more complex techniques in the Methods section.*
☐	☒	A description of all covariates tested
☐	☒	A description of any assumptions or corrections, such as tests of normality and adjustment for multiple comparisons
☐	☒	A full description of the statistical parameters including central tendency (e.g. means) or other basic estimates (e.g. regression coefficient) AND variation (e.g. standard deviation) or associated estimates of uncertainty (e.g. confidence intervals)
☐	☒	For null hypothesis testing, the test statistic (e.g. *F*, *t*, *r*) with confidence intervals, effect sizes, degrees of freedom and *P* value noted<br>*Give P values as exact values whenever suitable.*
☒	☐	For Bayesian analysis, information on the choice of priors and Markov chain Monte Carlo settings
☒	☐	For hierarchical and complex designs, identification of the appropriate level for tests and full reporting of outcomes
☒	☐	Estimates of effect sizes (e.g. Cohen's *d*, Pearson's *r*), indicating how they were calculated

Our web collection on statistics for biologists contains articles on many of the points above.

Software and code

Policy information about availability of computer code

Data collection	REDCap databases were used to record and store clinical metadata.
Data analysis	All analyses were performed using R version 4.3.1. The statistical files and scripts used for data analyses are also publicly available (https://github.com/mskelly7/COVID_RNASeq_Age).

For manuscripts utilizing custom algorithms or software that are central to the research but not yet described in published literature, software must be made available to editors and reviewers. We strongly encourage code deposition in a community repository (e.g. GitHub). See the Nature Portfolio guidelines for submitting code & software for further information.

Data

Policy information about availability of data

All manuscripts must include a data availability statement. This statement should provide the following information, where applicable:

- Accession codes, unique identifiers, or web links for publicly available datasets
- A description of any restrictions on data availability
- For clinical datasets or third party data, please ensure that the statement adheres to our policy

The RNA sequencing dataset supporting the conclusions of this study is available in the Gene Expression Omnibus (accession number: GSE231409; https://www.ncbi.nlm.nih.gov/geo/query/acc.cgi?acc=GSE231409). The sequencing dataset used for SARS-CoV-2 lineage assignment is available in the Sequence Read Archive (PRJNA1024980; https://www.ncbi.nlm.nih.gov/sra/?term=PRJNA1024980). The de-identified clinical metadata file is available at: https://github.com/mskelly7/COVID_RNASeq_Age/blob/main/Data_Files/BRAVE_RNASeq_Metadata.csv. Source data are provided with this paper.

Research involving human participants, their data, or biological material

Policy information about studies with human participants or human data. See also policy information about sex, gender (identity/presentation), and sexual orientation and race, ethnicity and racism.

Reporting on sex and gender	The study team recorded the self-reported sex at birth for all study participants. Gender was not collected as part of this study. RNAseq analyses were adjusted for self-reported sex at birth.
Reporting on race, ethnicity, or other socially relevant groupings	The study team recorded self-reported race and ethnicity for all study participants; however, race and ethnicity were not included in our analyses. Race and ethnicity data are included in the publicly available clinical metadata file.
Population characteristics	The study population is described in Table 1 of the manuscript. The study included a total of 202 participants, (age range of 1 week to 83 years), including 137 non-hospitalized individuals with mild SARS-CoV-2 infection and 65 healthy individuals. None of the individuals were vaccinated and none received anti-viral therapy. 51 of the infected individuals were classified as asymptomatic; 86 infected individual reported symptoms.
Recruitment	The samples and data included in this analysis were collected from participants in two studies conducted within the Duke University Health System (DUHS): the Biorepository of RespirAtory Virus Exposed (BRAVE) Kids study, which recruited SARS-CoV-2-exposed children and adolescents less than 21 years of age, and the Molecular and Epidemiological Study of Suspected Infection (MESSI) study, which recruited SARS-CoV-2-exposed adults 21 years of age of older. Participants were identified either through presentation to the health system for SARS-CoV-2 testing or through identification of a close contact with PCR-confirmed SARS-CoV-2 infection. Of note, we did not recruit participants with severe disease who required hospitalization. Participants in both studies were identified through review of SARS-CoV-2 testing conducted in the DUHS, and the study teams additionally approached close contacts of index cases for study participation. All participants included in this analysis were recruited between April 1, 2020, and December 31, 2020. None of the participants had a known SARS-CoV-2 infection prior to the current illness, nor had participants received a COVID-19 vaccine at the time of enrollment.
Ethics oversight	The relevant protocols were approved by the Duke University Health System (DUHS) Institutional Review Board (Pro00106150). Informed consent was obtained from all study participants or their legal guardians, with written approval obtained using an electronic consent document. The Molecular and Epidemiological Study of Suspected Infection (MESSI) study was approved by the DUHS Institutional Review Board (Pro00100241). Informed consent was obtained from all study participants, with written approval obtained using an electronic consent document. All study protocols were conducted in accordance with the Declaration of Helsinki, applicable regulations, and local policies.

Note that full information on the approval of the study protocol must also be provided in the manuscript.

Field-specific reporting

Please select the one below that is the best fit for your research. If you are not sure, read the appropriate sections before making your selection.

☒ Life sciences ☐ Behavioural & social sciences ☐ Ecological, evolutionary & environmental sciences

For a reference copy of the document with all sections, see nature.com/documents/nr-reporting-summary-flat.pdf

Life sciences study design

All studies must disclose on these points even when the disclosure is negative.

Sample size	The study described herein was an epidemiologic investigation of SARS-CoV-2 exposure and infection in among children and adults in central North Carolina. Individuals were identified through presentation to the health system for SARS-CoV-2 testing or through identification of a close contact with PCR-confirmed SARS-CoV-2 infection. No statistical method was used to predetermine sample size, experiments were not randomized, and the investigators were not blinded.
Data exclusions	Participants who tested negative for SARS-CoV-2 but reported one or more symptoms at enrollment or during study follow-up were excluded

	from analysis due to the potential for false-negative SARS-CoV-2 PCR results or infection with other adventitious agents. No other data were excluded from the analyses.
Replication	Not applicable - samples were collected from participants at a single timepoint and evaluated by bulk RNA sequencing. All sequencing runs included relevant positive and negative controls.
Randomization	Not applicable. The study was an epidemiologic evaluation of SARS-CoV-2 infection and exposure.
Blinding	Blinding was not relevant to the study, as this was an epidemiologic evaluation of SARS-CoV-2 infection and exposure. Investigators could not be blinded to the infection status of the participants.

Reporting for specific materials, systems and methods

We require information from authors about some types of materials, experimental systems and methods used in many studies. Here, indicate whether each material, system or method listed is relevant to your study. If you are not sure if a list item applies to your research, read the appropriate section before selecting a response.

Materials & experimental systems

n/a	Involved in the study
☒	☐ Antibodies
☒	☐ Eukaryotic cell lines
☒	☐ Palaeontology and archaeology
☒	☐ Animals and other organisms
☐	☒ Clinical data
☒	☐ Dual use research of concern
☒	☐ Plants

Methods

n/a	Involved in the study
☒	☐ ChIP-seq
☒	☐ Flow cytometry
☒	☐ MRI-based neuroimaging

Clinical data

Policy information about clinical studies

All manuscripts should comply with the ICMJEguidelines for publication of clinical research and a completedCONSORT checklist must be included with all submissions.

Clinical trial registration	The study was not a clinical trial and was not prospectively registered.
Study protocol	The study was not a clinical trial and was not prospectively registered.
Data collection	The samples and data included in this analysis were collected from participants in two studies conducted within the Duke University Health System (DUHS), located in central North Carolina, United States of America: the Biorepository of RespirAtory Virus Exposed (BRAVE) Kids study, which recruited SARS-CoV-2-exposed children and adolescents less than 21 years of age, and the Molecular and Epidemiological Study of Suspected Infection (MESSI) study, which recruited SARS-CoV-2-exposed adults 21 years of age of older. Recruitment in both studies included non-hospitalized participants who presented for SARS-CoV-2 testing within the health system and/or who had known close contact with an individual with confirmed SARS-CoV-2 infection (typically a household member). Participants in both studies were identified through review of SARS-CoV-2 testing conducted in the DUHS, and the study teams additionally approached close contacts of index cases for study participation. All participants included in this analysis were recruited between April 1, 2020, and December 31, 2020, prior to widespread circulation of major SARS-CoV-2 variants of concern. None of the participants had a known SARS-CoV-2 infection prior to the current illness, nor had participants received a COVID-19 vaccine at the time of enrollment.
Outcomes	Outcomes included SARS-CoV-2 infection status and the presence or absence of symptoms. Participants were considered to be infected with SARS-CoV-2 if the virus was detected by PCR testing of nasopharyngeal swabs collected for clinical or research purposes. The study team recorded all symptoms reported by the participants, and symptom questionnaires were administered to all participants Questionnaires were also administered to assess the presence and duration of specific symptoms at enrollment and at 7, 14, and 28 days after enrollment or until participants reported resolution of all symptoms. We evaluated for associations between the presence of specific symptom types and host responses derived from RNA sequencing.

Plants

Seed stocks	Not applicable.
Novel plant genotypes	Not applicable.
Authentication	Not applicable.
